# Cerebral amyloid angiopathy: an update

**DOI:** 10.1007/s00415-023-11631-3

**Published:** 2023-03-24

**Authors:** B. E. Schroeder, N. P. Robertson, T. A. T. Hughes

**Affiliations:** Department of Neurology, Division of Psychological Medicine and Clinical Neuroscience, Cardiff University, University Hospital of Wales, Heath Park, Cardiff, CF14 4XN UK

Cerebral amyloid angiopathy (CAA) is usually an age-related degenerative condition caused by deposition of beta-amyloid in the walls of cerebral small vessels. It is associated with micro-haemorrhages, lobar haemorrhages, focal superficial siderosis and changes in cerebral white matter. In addition to a range of persistent and fixed deficits, it can also be associated with transient symptoms commonly referred to as “amyloid spells”. As a result, urgent review in Transient Ischaemic Attack (TIA) clinics is common. However, the approach required for the diagnosis and management of these patients can differ substantially from the more traditional assessment offered to patients with common causes of suspected cerebral ischaemia.

The gold standard for diagnosis remains histopathological examination, although the Edinburgh and Boston criteria provide clinicians with a probabilistic approach for diagnosis largely based on imaging characteristics. In this month’s Journal Club, we review the latest version of the Boston criteria based on Magnetic Resonance Imaging (MRI), the Simplified Edinburgh criteria based on computed tomography (CT) and the use of complement as a potential biomarker in this disease.

## The Boston criteria version 2.0 for cerebral amyloid angiopathy: a multicentre, retrospective, MRI-neuropathology diagnostic accuracy study

The original Boston criteria for diagnosing CAA with MRI were first introduced in the 1990s, with a subsequent modified version in 2010. In this paper, the authors describe the use of MRI markers of disease together with gold standard histopathology to form version 2.0 of the Boston criteria by updating and externally validating the modified criteria across the full spectrum of presentations associated with CAA.

Clinical, radiological and histopathological data were used to determine specificity and sensitivity and optimised using MRI features in a prespecified derivation cohort (*n* = 159) seen in Massachusetts General Hospital between 1994 and 2012. The derived criteria were then externally validated in a temporal validation cohort (*n* = 59) identified between 2012 and 2018 together with a geographic validation cohort from other centres (*n* = 123) and compared to the currently employed (modified) Boston criteria with histopathological assessment as the gold standard.

From the derivation cohort, provisional criteria for probable CAA were identified and included: at least two strictly lobar haemorrhagic lesions (intracerebral haemorrhage, cerebral microbleeds or a focus of cortical superficial siderosis), or at least one strictly lobar haemorrhagic lesion combined with at least one white matter lesion (i.e. severe visible perivascular spaces in the centrum semiovale or white matter hyperintensities in a multispot pattern). For a diagnosis of probable CAA, the Boston 2.0 criteria were superior to the modified Boston criteria with an area under the curve (AUC) of 0.798 (95% CI 0.741–0.854, *p* = 0.0005), reasonable sensitivity 74·5% (65·4–82·4) and good specificity 95.0% (83.1–99.4) across all individuals who had autopsy as the diagnostic standard.

*Comment:* In patients aged 50 years and older, the Boston criteria version 2.0 incorporates emerging MRI markers to provide clinicians with a more sensitive, but easily applied tool for the diagnosis of CAA, a condition which is exposing the inadequacy of routine clinical assessment and CT imaging of the brain. Further work to identify the real-world sensitivity of the new criteria will be valuable in larger and more diverse populations but what is clear is the importance of MR imaging in older patients with transient neurological symptoms, lobar haemorrhage or cognitive impairment. The diagnostic issues are of particular relevance in those patients who have other co-morbidities such as atrial fibrillation and carotid atheroma, to which transient symptoms could be attributed and for which anti-platelets or anticoagulants may be prescribed.

Lancet Neurol. 2022 Aug;21(8):714–725.

## Simplified Edinburgh CT criteria for identification of lobar intracerebral haemorrhage associated with cerebral amyloid angiopathy

CT is more commonly available than MRI, especially in developing areas, and is effective in detecting the presence and distribution of significant intraparenchymal and subarachnoid haemorrhage, but does not always detect micro-haemorrhages or superficial siderosis. The Edinburgh criteria described in this paper stratify the probability of CAA (low, medium and high) in patients with lobar haemorrhages revealed by CT.

The authors identified 210 consecutive patients with both MR and CT imaging from a prospective cohort study of 614 patients, all of whom had CT imaging. Blinded neuroradiologists evaluated both CT and MR imaging. There were significant differences between those patients included and excluded, for example in hospital mortality (*p* < 0.001), modified Rankin Scale score (*p* < 0.001) and simplified Edinburgh CT criteria risk (*p* < 0.001). The inter-rater variability and diagnostic accuracy of the Edinburgh CT-based criteria for identification of ICH associated with CAA were assessed using the modified Boston criteria as a reference standard. Risk categories of the simplified Edinburgh criteria were as follows: low CAA risk, neither finger-like projections nor SAH; medium CAA risk, SAH only; high risk, both finger-like projections and SAH.

Of those individuals included (*n* = 210), 73 (34.8%) had a low risk, 67 (31.9%) a medium risk and 70 (33.3%) a high risk of CAA-associated ICH, with moderate inter-rater variability. The ability of the Edinburgh criteria to “rule in” (high-risk) had a high specificity 87.1% (95% CI 79.3–92.3) and positive likelihood ratio 4.5 (95% CI 2.7–7.5) but a sensitivity of only 58.5% (95% CI 47.8–68.4), whereas “rule out” (low risk) had a specificity of 47.4% (95% CI 38.1–56.9), negative likelihood ratio of 0.4 (95% CI 0.3–0.6) and sensitivity of 80.9% (95% CI 71.1–88.0).

*Comment*: The combination of finger-like projections and SAH (the high-risk category) showed clinical net benefit for ruling in probable CAA with a specificity of 87% according to the MRI-based modified Boston criteria but their high rule-out sensitivity (81%) was not associated with a clinical net benefit. For the general neurologist, the message seems to be that in the right clinical context without an obvious alternative diagnosis, a patient with finger-like projections and subarachnoid haemorrhage is likely to have CAA. The Edinburgh criteria do not include clinical information which, for a scale designed for clinicians without easy access to MR imaging, feels like a missed opportunity to enhance the clinical relevance of the scale.

As always, the clinical context is likely to determine practice regardless of the probabilities of diagnosis based on studies of groups of patients. In situations where important individual decisions on anticoagulation, driving, employment and prognosis hinge on the exact nature of an intracerebral haemorrhage, it is difficult to justify using CT imaging only. However, this study will inform prioritisation of patients for MR imaging and raise awareness of this important condition.

Neurology. 2022 May 17;98(20):e1997–e2004.

## Complement 3 is a potential biomarker for cerebral amyloid angiopathy

Complement activation has been implicated in CAA and elevated microglial C3 has been shown in patients with CAA relative to controls but it is unclear if this is cause or consequence.

This is a retrospective study of patients (*n* = 55, 60% males, mean (SD) age 76.3 (6.8) years) with mild cognitive impairment who had been screened for a treatable cause in a single stroke centre. Patients with dementia were excluded. Probable or possible CAA was diagnosed using MR imaging according to the modified Boston criteria; four (7%) and 12 (22%) had possible or probable CAA, respectively. Blood sera were gathered within 6 months of cognitive assessment. C3 levels were evaluated by sandwich enzyme-linked immunoassay.

Comparison of CAA to non-CAA sera demonstrated a significant difference in C3 titre—CAA: 0.43 u/mL [0.34–0.65] vs non-CAA: 0.35 u/mL [0.25–0.45], *p* = 0.040. Levels were similar between probable and possible CAA and C-reactive peptide levels were similar across all groups (*p* = 0.955).

Univariate logistic regression identified elevated C3 level and absence of lacunar infarcts to be predictive of CAA. Stepwise selection with backward elimination (*p* threshold = 0.1) maintained these two variables as independent predictors (odds ratio [95% CI])—C3 levels: 1.407 [1.042–1.899], *p* = 0.026; lacunar infarcts: 0.203 [0.044–0.925], *p* = 0.039). C3 levels alone had an Area Under Curve of Receiver Operating Characteristic (AUC-ROC) of 0.68 (0.53–0.83) for predicting CAA.

*Comment:* The authors show that C3 titres are elevated in patients with CAA compared to mild cognitive impairment controls in a small retrospective study. Validation on larger cohorts will be valuable in assessing this new potential biomarker. Inclusion of patients with dementia will also provide more real-world utility as amyloid deposition is a common feature of Alzheimer’s disease.

J Alzheimers Dis. 2022;89(1):381–387.

## Conclusion

Clinical practice in cerebrovascular disease is changing. It has been revolutionised by advances in preventative and acute treatments with numbers needed to treat of the order of approximately 100 with aspirin after a stroke, 10 for thrombolysis and carotid endarterectomy and 2–3 for thrombectomy. The idea that anti-platelets (RESTART trial) and anticoagulation (ENRICH-AF) may not necessarily be harmful for patients with a history of intracerebral haemorrhage is overturning long-held beliefs and assumptions.

Cerebral amyloid angiopathy acts as both cheerleader and scaremonger of the conditions challenging old practices, as it forces clinicians to broaden their differential diagnosis when confronted with patients with transient and persistent neurological deficits. CAA has a varied repertoire of clinical presentations many of which can make clinicians ask the wrong questions or prescribe inappropriate medications. The papers described here make the case for MR imaging in suspected cases of CAA and the Edinburgh criteria will help with prioritisation.

